# Comparative Genomics of Seasonal Senescence in Forest Trees

**DOI:** 10.3390/ijms23073761

**Published:** 2022-03-29

**Authors:** Anastasia Y. Batalova, Yuliya A. Putintseva, Michael G. Sadovsky, Konstantin V. Krutovsky

**Affiliations:** 1 Department of Genomics and Bioinformatics, Institute of Fundamental Biology and Biotechnology, Siberian Federal University, 660041 Krasnoyarsk, Russia; batalova0910@mail.ru; 2 Department of Biophysics, Institute of Fundamental Biology and Biotechnology, Siberian Federal University, 660041 Krasnoyarsk, Russia; yaputintseva@mail.ru; 3 Institute of Computational Modelling, Russian Academy of Sciences, Siberian Branch, 660036 Krasnoyarsk, Russia; msad@icm.krasn.ru; 4 V. F. Voino-Yasenetsky Krasnoyarsk State Medical University, 660022 Krasnoyarsk, Russia; 5 Federal Siberian Research Clinical Center, Federal Medical-Biological Agency, 660037 Krasnoyarsk, Russia; 6 Department of Forest Genetics and Forest Tree Breeding, Georg-August University of Göttingen, 37077 Göttingen, Germany; 7 Center for Integrated Breeding Research, Georg-August University of Göttingen, 37075 Göttingen, Germany; 8 Laboratory of Population Genetics, N. I. Vavilov Institute of General Genetics, Russian Academy of Sciences, 119333 Moscow, Russia; 9 Scientific and Methodological Center, G. F. Morozov Voronezh State University of Forestry and Technologies, 394087 Voronezh, Russia

**Keywords:** *Larix sibirica*, seasonal leaf senescence, comparative genomics, forest trees

## Abstract

In the course of evolution, both flowering plants and some gymnosperms have developed such an adaptation to winter and unfavorable living conditions as deciduousness. Of particular interest is Siberian larch (*Larix sibirica* Ledeb.), which is the only species in the pine family (Pinaceae) with a seasonal deciduousness. New generation sequencing technologies make it possible to study this phenomenon at the genomic level and to reveal the genetic mechanisms of leaf and needle aging in angiosperms and gymnosperms. Using a comparative analysis of the genomes of evergreen and deciduous trees, it was found that the genes that control EXORDIUM LIKE 2 (EXL2) and DORMANCY-ASSOCIATED PROTEIN 1 (DRM1) proteins are most represented in Siberian larch, while an excess of genes that control proteins acting as immune receptors were found in evergreens. Orthologs from the family of genes that control leucine-rich repeat receptor-like kinases (LRR-RLK) contributed mostly to the distinction between evergreens and deciduous plants.

## 1. Introduction

Deciduousness and seasonal senescence are evolutionarily important adaptations to winter and unfavorable living conditions in higher plants (both angiosperms and gymnosperms). Genetic regulation of the seasonal senescence is of primary interest for us. Deciduousness in broad-leaved woody plants of the boreal zone is clearly related to the autumn season: the vast majority of boreal plant species lose their foliage at the end of the vegetation season. Obviously, this reaction is strongly correlated with weather and is regulated by environmental factors such as temperature and daylight length. In cold winter, most perennials hibernate and shed their foliage entirely. Simultaneously, the metabolism of these plants changes and slows down.

The majority of conifer species in the boreal climatic zone are evergreen. Needles fall off throughout the entire year with an intensity that does not have a pronounced seasonality. It is typical for evergreens and the vast majority of conifers, which are gymnosperms. Larches in the Genus *Larix*, including *Larix sibirica* Ledeb. represent an exception among conifers, shedding all the needles in the fall, like most flowering plants do.

### 1.1. Genetic Regulation of Leaf Senescence

The genes controlling proteins of the NAC and WRKY families are among the most common and well-studied genetic regulators of leaf senescence [1]. Expression of these genes depends on both internal (changes in the level of reactive oxygen species (ROS), PH, metal ions, and hormones) and external (drought, cold, and light level) signals [1].

In addition, the onset of the leaf aging stage depends on the rate of development of the leaves themselves from the moment of their formation. Coordination of the stages of leaf development is associated with a number of physiological processes and associated with them are gene expression such as cell growth and proliferation (genes *BOP1, KNAT1, TCP4, miR396, GPFs, GIFs, ARGOS, ANT,* and *TOR*), hormonal regulation associated with cytokinins (*AHKs* and *CRFs*) and auxins (*ARF2* and *SAUR36*), transmission of signals of oxidative stress (*FHY3*, *FAR1* and *REF*), light transmission of signals (*phyA*, *PHYb*, *PIFs,* and *GLK2*), and circadian regulation (*TOC1*) [1].

Gene expression can be regulated at several levels: transcriptional, post-transcriptional, translational, and post-translational.

#### 1.1.1. Regulation at the Transcriptional Level

The regulation of gene expression at the transcriptional level is carried out mainly by modifying histones and enzymes that model chromatin. It has been shown that mutations in the helicase domain in SWI2/SNF2-like chromatin-remodeling proteins, DRD1 and DDM1, delay leaf senescence in *Arabidopsis thaliana* and also increase its lifespan [2]. Normally, these proteins, participating with ATP, change the composition and arrangement of nucleosomes, allowing other proteins to gain access to DNA, in particular DNA methyltransferases, which is necessary for suppressing gene expression (silencing). Histone H3K4 demethylase JMJ16 negatively regulates leaf senescence in *Arabidopsis*, at least partly through repressing the expression of positive regulators of leaf senescence, *WRKY53* and *SAG201* [3].Yellowing of leaves due to the destruction of chlorophyll is one of the visible manifestations of leaf aging. Chlorophyll degrades with the participation of EIN3, ANAC092 (ethylene-mediated degradation) [4] and MYC2/3/4 (in the presence of methyl jasmonate) genes [5]. The transcription factor ANAC046 provides a positive feedback in the regulation of leaf senescence; it directly binds to promoters of genes associated with chlorophyll catabolism (*NYC1*, *NYE*, and *NYE2*), activating their expression [6]. The genes *ANAC042*, *ANAC017/082/090*, *ANAC083*, *WRKY54/70*, *WRKY18/40/60*, *bHLH03/13/14/17*, *MYB44*, *LUX*, *TCP19*, and *TCP20* inhibit the leaf senescence [1].

#### 1.1.2. Regulation at the Post-Transcriptional Level

Several studies reported post-transcriptional regulation of leaf senescence by some miRNAs [7,8,9,10]. For instance, *miR164* expression gradually decreases with leaf aging through negative regulation by *EIN2* [7], which leads to the elaborate up-regulation of *ORE1* expression (a positive regulator of cell death and leaf senescence in *Arabidopsis*) [8]. Plants with over-expression of miR319 exhibit the phenotype of delayed leaf senescence; the target is *TCP* genes that play the key role in leaf senescence [9]. MiR390 activates the production of tasiRNAs (formed from TAS3) targeted on *ARF2* gene (positive regulator of leaf senescence) [10].

Mutations in the *HDA9* (histone deacetylase 9) gene slow down leaf aging [11]. It was suggested that HDA9 in complex with a SANT domain-containing protein POWERDRESS (PWR) (that facilitates the transport of HDA9 from the cytoplasm into the nucleus) and transcription factor WRKY53 (that attaches to the W-box) binds the promoters of the key negative regulators of aging (*APG9*, *NPX1*, and *WRKY57*) and then deacetylates lysine residues at the *n*-terminal portion of histones, thereby suppressing gene expression.

#### 1.1.3. Regulation at the Translational Level

A mutation in the *ORE4* gene is known for the delay of senescence and the growth rate of leaves [12]. It was suggested that it is associated with a decrease in the photosynthesis rate due to inhibiting the expression of the *PRPSI7* gene. Interestingly, the mutation delayed natural aging, but it had a minor effect on aging induced by darkness, abscisic acid (ABA), methyl jasmonate (MeJA), and ethylene.

#### 1.1.4. Regulation at the Post-Translational Level

Post-translational regulation involves phosphorylation, glycosylation, ubiquitylation, methylation, and acetylation that affect the conformation, activity, stability, and localization of proteins [13]. WRKY53 enzyme provides an example of post-translational regulation; it is a positive regulator of leaf senescence. This enzyme can be phosphorylated by MEKK1 (mitogen-activated protein kinase kinase kinase 1), resulting in the growth of its ability to bind to target promoters in DNA [14]. It is deactivated due to ubiquitination of the WRKY53 protein by ubiquitin ligase UPL5 [15]. RECEPTOR PROTEIN KINASE 1 (RPK1) has an important regulatory role in ABA-mediated and age-dependent leaf senescence. Loss-of-function mutations in RPK1 lead to a significant delay in ABA-induced and age-dependent leaf senescence [16].

### 1.2. The Role of Phytohormones in the Regulation of Leaf Senescence

With the age of leaves, the content of some phytohormones (ethylene, jasmonic acid, salicylic acid) in them increases, while others (gibberellic acid, cytokinins, and auxins) decrease. It is believed that the first group of phytohormones promotes leaf aging, while the second delays their aging [17]. It was shown that mutation in the *ARF2* gene, a transcription factor associated with the response to auxin, delayed leaf senescence [18]. The following genes are known to promote leaf aging: *EIN2*, *EIN3*, *EIL1* (ethylene signaling pathway), *COI1*, *MYC2* (jasmonic acid signaling pathway), *ICS1*, *NPR1*, *EDS1*, *PAD4* (salicylic acid signaling pathway), *PYL9*, *SnRK2.8*, *ABI5* (abscisic acid signaling pathway), *TiR1*, *ARF2* (part of the auxin signaling pathway); *BRI1* (brassinosteroid control) [17]. Genes that slow down leaf aging include *JAZ7* (jasmonic acid), *DELLAS* (gibberlins), *AHK3*, and *ARR2* (cytokinins) [17].

## 2. Results

The number of eukaryotic and undefined orthologs selected by the EggNOG program in the conifer genomes for the comparative analysis are presented in Table 1.

**Figure 1:**
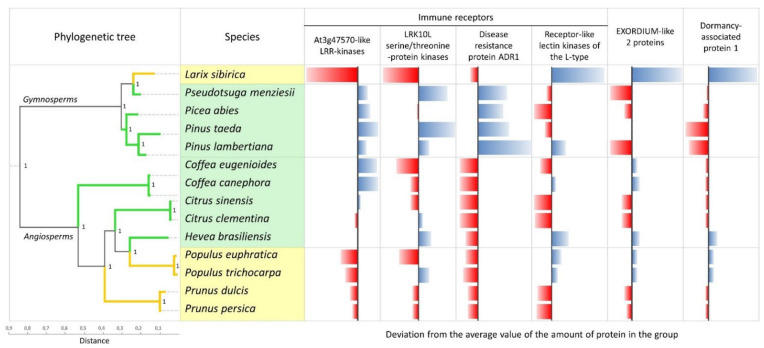
Quantitative analysis of the representation of protein groups in the studied organisms performed using the OrthoFinder program. Yellow and green colors highlight deciduous and evergreen species, respectively. The multigenic phylogenetic tree was obtained using the STAG method for inferring a species tree from a set of single gene trees (supertree approach). Red color highlights less than the average number of proteins in the group, blue highlights more.

Six orthogroups were found with a statistically significant quantitative difference in the number of orthologs within an orthogroup between evergreen and deciduous trees based on the two-sample *t*-test (Figure 1, Table 2).

The first four groups are immune receptors, while the last two are sugar-associated: EXORDIUM-like 2 is involved in sugar sensing, and dormancy-associated protein 1 is involved in responses to fructose, glucose, and sucrose. A smaller number of proteins were observed in *L. sibirica* than in evergreen conifers in the first three groups, and it was the opposite in the next three groups. In the fourth group, the protein value in *Pinus lambertiana*, unlike other conifers, was slightly above average, similar to *L. sibirica*, but still significantly lower than in *L. sibirica* (*p* = 4.2 × 10^−6^, Table 3). It is important to note that in the first group, the pattern of protein distribution in deciduous and evergreens was the same for both gymnosperms and angiosperms—the number of proteins was much lower in deciduous species than in evergreens. An orthogroup is a set of genes from multiple species descended from a single gene in the last common ancestor (LCA) of that set of species [19]. There were orthogroups presented in only a few species, but we selected only those that were present in all organisms. However, the remaining orthogroups that are represented in only a few species are also of interest for further research. In general, the generated phylogenetic tree in Figure 1 is in agreement with consensual phylogenetic relationships for these species based on traditional markers [20,21].

The OrthoFinder software was used to carry out a comparative analysis of the proteomes of evergreen and deciduous trees. As a result, a difference was found in the representation of orthologs associated with cell reception and signaling. The common domain for these proteins was a protein kinase domain (“Pkinase”; identifier in the PFAM database-PF00069). Among the proteins containing the “Pkinase” domain, 1496 orthogroups were identified.

We additionally tested under- and overrepresentation of ortholog members in the six selected orthogroups for conifers using Fisher’s exact test for a 2 × 2 contingency table. The obtained two-tailed *p*-values that supported the selection of these orthogroups that demonstrated under- or overrepresentation of ortholog members in deciduous *L. sibirica* vs. all other four evergreen conifer species (Table 3).

**Figure 2:**
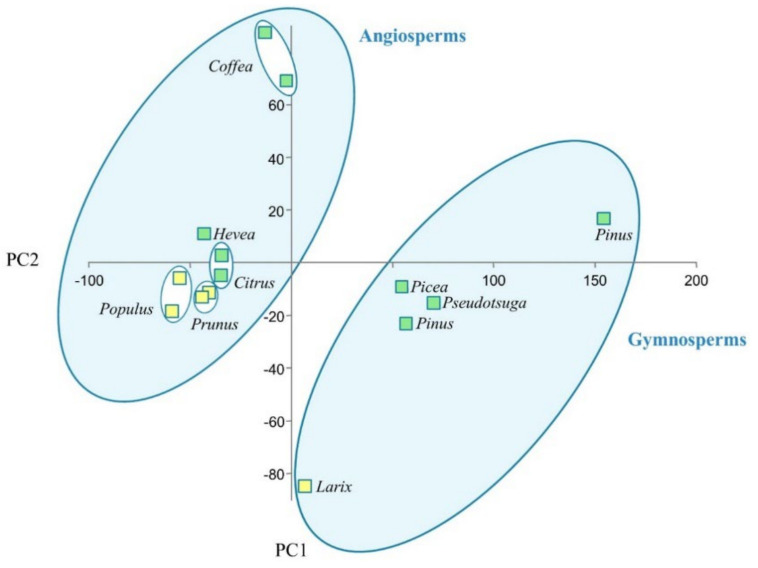
Cluster analysis of orthogroups containing proteins of the protein kinase family by principal component analysis (PCA) based on Euclidean distance using Past3 v. 3.22 software. Green squares represent evergreen species, and yellow squares represent deciduous.

Principal components analysis showed that for proteins containing the protein kinase domain (Table 4) the angiosperms and gymnosperms formed two separate clusters (Figure 2). In the angiosperms cluster, a certain order in the mutual arrangement of the studied taxa is revealed. In general, evergreens are located in the upper half of the cluster, while deciduous ones are in the lower half. A similar pattern is observed in the cluster of gymnosperms: the only deciduous species (Siberian larch) occupies a separate position in this cluster. In general, order of mutual arrangement of taxa according to deciduousness was similar in angiosperms and gymnosperms.

**Figure 3:**
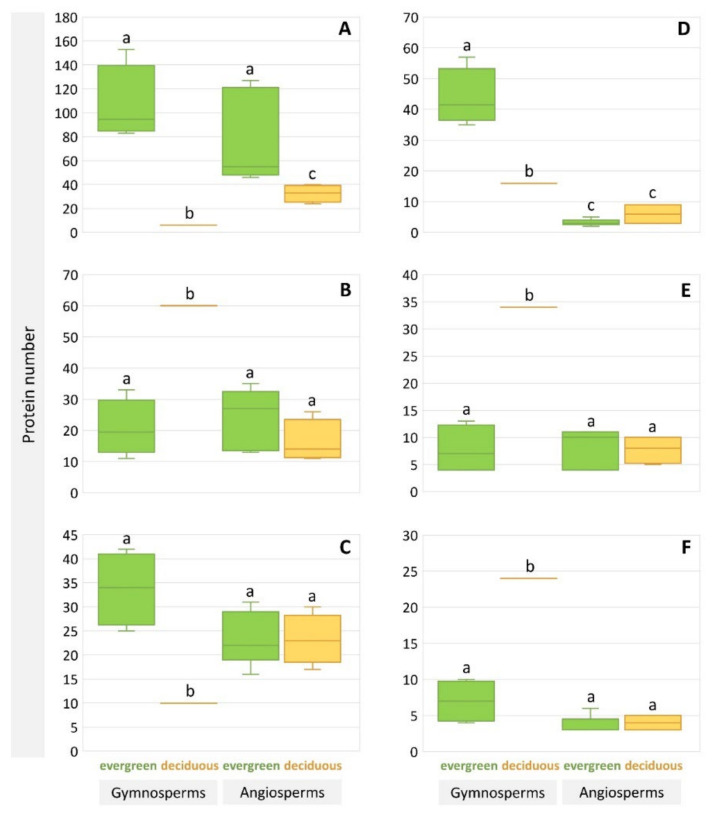
Analysis of the representation of orthologs: (**A**)-At3g47570-like LRR-kinases, (**B**)-receptor-like lectin kinases of the L-type, (**C**)-LRK10L serine/threonine-protein kinases, (**D**)-disease resistance protein ADR1, (**E**)-EXORDIUM-like 2 proteins, (**F**)-DRM1-like proteins. Statistically significant differences are shown using the letters a, b, and c. A common letter means no difference. Yellow and green colors highlight deciduous and evergreen species, respectively. Different letters represent statistically significant difference at *p* < 0.05 based on the two-sample *t*-test.

The differences in the representation of some orthologues between evergreen and deciduous forest trees are shown in Figure 3. Evergreens, both gymnosperms and angiosperms, differed from deciduous species in the representation of At3g47570-like LRR-kinases orthologues (Figure 3A). For other orthogroups among angiosperms, there were no differences between deciduous and evergreen species (Figure 3B–F). However, peculiarities were found in gymnosperms, namely: *L. sibirica* had more proteins in the orthogroups of receptor-like lectin kinases of the L-type (Figure 3B), EXORDIUM-like 2 proteins (Figure 3E), and DRM1-like proteins (Figure 3F); while evergreens had a higher representation of proteins in the orthogroups LRK10L serine/threonine-protein kinases (Figure 3C) and disease resistance protein ADR1 (Figure 3D).

## 3. Discussion

The orthologues of the At3g47570 *Arabidopsis* gene, encoding serine/threonine kinase-like proteins containing leucine repeats contributed mostly into the distinction between evergreens and deciduous plants (Figure 3A). This observation is in agreement with published data on the increase in activity of this kinase with the leaf age in *Arabidopsis thaliana* [22]. The At3g47570 gene is also a target of the WRKY40 transcription factor, which is involved in the regulation of the protective response when exposed to pathogenic microflora [23].

For Siberian larch, an enriched cluster was found that comprises the orthologs of the receptor-like L-type lectin kinase (LecRK) (Figure 3B). The proteins of this type are involved in important biological process of the protection from pathogens [24]. It was reported that overexpression of LecRK-IX.1 or LecRK-IX.2 in *Arabidopsis* increased *Phytophthora* resistance but also induced cell death [25]. However, cell death induced by overexpression of LecRK-IX.1 and LecRK-IX.2 was not correlated with leaf senescence [25].

Contrary to deciduous conifers, evergreen conifers are characterized by a predominance of orthologs of the receptor-like serine/threonine protein kinase LRK-10L (biological process-innate cellular response) (Figure 3C). The loss-of-function mutants of the *Arabidopsis* orthologue of the wheat LRK10 gene showed ABA-insensitive and drought stress-sensitive phenotypes [26].

The difference between deciduous and evergreen conifers in the abundance of disease resistance associated proteins (LRR-kinases, LRK10L serine/threonine-protein kinases, ADR1 and receptor-like lectin kinases of the L-type) was also found (Table 3). Additionally, they are all immune receptors. ADR1 proteins contain NB-ARC domain, and it is known that NB-ARC proteins function as molecular switches regulating many processes, including immunity and apoptosis [4]. Resistance (R) genes are associated with preformed and inducible defenses [27]. It was also suggested that R genes likely contribute to the remarkable longevity of *Ginkgo biloba* [28]. ADR1 orthologues are most represented in evergreen conifers (Figure 3D). They convey broad spectrum disease resistance. Overexpression of ADR1 also increased drought tolerance in *Arabidopsis* [29].

In the reconstructed proteome of larch, the orthologs of the EXL2 (Figure 3E) and DRM1 (Figure 3F) proteins were more abundant in comparison to evergreen trees. The EXL2 protein is involved in the signaling pathway of sugars; an increase in the expression of this protein occurs with a decrease in the availability of carbon [30]. The activity of the dormancy-associated DRM1 protein increases during leaf senescence and is associated with the response to the level of glucose, sucrose, and fructose [31].

## 4. Materials and Methods

Genomes and their annotations for 14 studied species were downloaded from the open NCBI GenBank database (https://www.ncbi.nlm.nih.gov. accessed on 1 February 2020) and presented in Table 5, including the genome of *L. sibirica* [32].

Extraction of coding nucleotide sequences of genes in the studied genomes was carried out using Cufflinks software package v. 2.2.1 [33]. Then, the retrieved sequences were translated into amino acids using EMBOSS software v. 6.6.0 [34].

We double-checked the plant genomes used in our study to exclude foreign sequences representing bacteria, viruses, fungi, etc., from the analysis of the plant specific genes. Subsequently, all nucleotide sequences encoding proteins isolated from the assembled genomes were checked against genes representing archaea, bacteria, eukaryotes, and viruses using EggNOG-mapper v. 2.1.6 [35] to strictly select only the eukaryotic orthologs for further analysis and also undefined orthologs that do not belong archaea, bacteria, and viruses. Annotation of protein sequences by functional domains was performed using InterProScan software v. 79.0 [36].

The orthologs search was performed using OrthoFinder software v. 2.3.12 [19]. For the analysis of protein data, we used with default parameters DIAMOND for sequence similarity search [37], *mafft* for multiple sequence alignment, and *fasttree* for tree inference using a maximum likelihood approach with the JTT model [38]. The multigenic phylogenetic tree was obtained using the STAG method for inferring a species tree from a set of single gene trees (supertree approach) [39] and was visualized using Dendroscope 3 software v. 3.7.2 [40,41]. Upon clustering of the proteins into orthologous groups, the orthogroups by which *L. sibirica* differed from other conifers were analyzed using the BLAST program v. 2.11.0 in the UniProt database [42]. All genes in an orthogroup were supposedly descended from a single ancestral gene [27]. Principal component analysis (PCA) was performed to cluster orthogroups using the Past3 software v. 3.22 [43] and pairwise Euclidean distances based on differences in the representation of genes in orthogroups.

## 5. Conclusions

The presented data show that the proteins functioning as immune receptors are the most abundant in evergreen trees. They also provide resistance to pathogens and may also be associated to the longer life expectancy of conifers. It supports the role of immune defense in the regulation of leaf lifespan. The orthologues of the LRR receptor-like serine/threonine-protein kinase At3g47570 made the greatest contribution to the difference between evergreen and deciduous plants, which is in agreement with published data on their activity increase during leaf senescence in *Arabidopsis thaliana*, whereas other orthogroups in *L. sibirica* were also very significantly different in number than others. It is unlikely that studying more species would change the main findings and conclusions, but certainly additional species should be studied and can provide more details on genetic differences between evergreen and deciduous.

## Figures and Tables

**Table 1:** Number of the eukaryotic and undefined orthologs used for the comparative analysis in this study.

Group	Type	Species	Eukaryotes	Undefined	Total
Gymnosperms (Pinaceae)	deciduous	*Larix sibirica*	34,323	4391	38,714
	evergreen	*Pseudotsuga menziesii*	46,066	478	46,544
		*Pinus taeda*	51,113	527	51,640
		*Pinus lambertiana*	38,024	436	38,460
		*Picea abies*	43,626	-	43,626
Angiosperms (Pentapetalae)	deciduous	*Populus euphratica*	30,272	414	30,686
		*Populus trichocarpa*	31,209	424	31,633
		*Prunus persica*	22,706	425	23,131
		*Prunus dulcis*	22,765	-	22,765
	evergreen	*Coffea eugenoides*	27,514	1584	29,098
		*Coffea canephora*	23,204	2344	25,548
		*Hevea brasiliensis*	34,297	867	35,164
		*Citrus sinensis*	24,119	412	24,531
		*Citrus clementina*	22,523	296	22,819

**Table 2:** Number of genes in six orthogroups in the genomes of evergreen and deciduous trees.

Type	Species	At3g47570-Like LRR-Kinases	LRK10L Serine/Threonine-Protein Kinases	Disease Resistance Protein ADR1	Receptor-Like Lectin Kinases of the L-Type	EXORDIUM-Like 2 Proteins	Dormancy-Associated Protein 1	Total
deciduous	*Larix sibirica*	6	10	9	60	34	24	38,714
evergreen	*Pseudotsuga menziensii*	90	38	31	20	4	10	46,544
	*Pinus taeda*	153	42	32	11	13	4	51,640
	*Pinus lambertiana*	83	30	46	19	4	5	38,460
	*Picea abies*	99	25	29	33	10	9	43,626
deciduous	*Populus euphratica*	24	17	6	16	10	5	30,686
	*Populus trichocarpa*	29	30	5	26	10	5	31,633
	*Prunus persica*	40	23	8	11	6	3	23,131
	*Prunus dulcis*	37	23	8	12	5	3	22,765
evergreen	*Coffea eugenoides*	115	16	2	35	10	3	29,098
	*Coffea canephora*	127	22	2	30	11	3	25,548
	*Hevea brasiliensis*	50	31	6	27	11	6	35,164
	*Citrus sinensis*	55	22	3	14	4	3	24,531
	*Citrus clementina*	46	27	3	13	4	3	22,819

**Table 3:** Probability (*p*) values of under-(↓) or over-(↑) representation of ortholog members in the orthogroups estimated using Fisher’s exact test for a 2 × 2 contingency table.

Orthogroup	Species	*Larix sibirica*	*Pseudotsuga menziensii*	*Pinus taeda*	*Pinus lambertiana*
At3g47570-like LRR-kinases	*Pseudotsuga menziesii*	2.2 × 10^−16^↓			
	*Pinus taeda*	2.2 × 10^−16^↓	0.001↓		
	*Pinus lambertiana*	2.2 × 10^−16^↓	0.492	0.021↑	
	*Picea abies*	2.2 × 10^−16^↓	0.276	0.043↑	0.766
LRK10L serine/threonine-protein kinases	*Pseudotsuga menziesii*	6.8 × 10^−4^↓			
	*Pinus taeda*	4.0 × 10^−4^↓	1.000		
	*Pinus lambertiana*	1.4 × 10^−3^↓	0.903	0.906	
	*Picea abies*	0.041↓	0.207	0.178	0.281
Disease resistance protein ADR1	*Pseudotsuga menziesii*	3,8 × 10^−3^↓			
	*Pinus taeda*	6,8 × 10^−3^↓	0.802		
	*Pinus lambertiana*	2,4 × 10^−^^7^↓	0.012↓	0.004↓	
	*Picea abies*	5,0 × 10^−3^↓	1.000	0.798	0.015↑
Receptor-like lectin kinases of the L-type	*Pseudotsuga menziesii*	1.1 × 10^−7^↑			
	*Pinus taeda*	6.6 × 10^−13^↑	0.071		
	*Pinus lambertiana*	4.2 × 10^−6^↑	0.748	0.026↓	
	*Picea abies*	8.1 × 10^−4^↑	0.053↓	0.000↓	0.164
EXORDIUM-like 2 proteins	*Pseudotsuga menziesii*	1.9 × 10^−8^↑			
	*Pinus taeda*	5.0 × 10^−5^↑	0.054↓		
	*Pinus lambertiana*	6.0 × 10^−7^↑	1.000	0.142	
	*Picea abies*	5.6 × 10^−5^↑	0.109	1.000	0.192
DRM1-like proteins	*Pseudotsuga menziesii*	5.0 × 10^−3^↑			
	*Pinus taeda*	3.8 × 10^−6^↑	0.106		
	*Pinus lambertiana*	5.5 × 10^−4^↑	0.442	0.510	
	*Picea abies*	4.5 × 10^−3^↑	1.000	0.102	0.437

**Table 4:** Representation of functional protein domains.

Species	NB-ARC (PF00931)	Phi 1 (PF04674)	Oxidored FMN (PF00724)	Pkinase (PF00069)	AP2 (PF00847)	ABA-WDS (PF02496)	14-3-3 (PF00244)	Bet v 1 (PF00407)
*Larix sibirica*	136	66	48	1867	374	46	26	63
*Pseudotsuga menziensii*	508	22	34	1920	139	5	13	35
*Pinus taeda*	849	27	18	2482	190	1	3	48
*Pinus lambertiana*	780	16	28	1750	125	8	4	52
*Picea abies*	696	41	28	1930	269	16	22	49
*Populus euphratica*	327	15	4	1501	216	1	16	47
*Populus trichocarpa*	566	16	9	1228	205	3	18	50
*Prunus persica*	388	10	10	1050	126	3	14	44
*Prunus dulcis*	377	10	7	1046	129	4	13	46
*Coffea eugenoides*	948	13	13	1398	133	4	15	57
*Coffea canephora*	798	15	18	1281	104	3	15	47
*Hevea brasiliensis*	575	17	11	1931	215	5	26	62
*Citrus sinensis*	615	7	9	1180	136	2	18	35
*Citrus clementina*	578	7	10	1083	125	3	15	32

**Table 5:** Genomes of 14 studied species.

Type		Species	NCBI GenBank #
Gymnosperms	deciduous	*Larix sibirica*	GCA_004151065.1
	evergreen	*Pseudotsuga menziensii*	GCA_001517045.1
		*Pinus taeda*	GCA_000404065.3
		*Pinus lambertiana*	GCA_001447015.2
		*Picea abies*	GCA_900491625.1
Angiosperms	deciduous	*Populus euphratica*	GCA_000495115.1
		*Populus trichocarpa*	GCA_000002775.3
		*Prunus persica*	GCA_000346465.2
		*Prunus dulcis*	GCA_902201215.1
	evergreen	*Coffea eugenoides*	GCA_003713205.1
		*Coffea canephora*	GCA_900059795.1
		*Hevea brasiliensis*	GCA_001654055.1
		*Citrus sinensis*	GCA_000317415.1
		*Citrus clementina*	GCA_000493195.1

## Data Availability

All data are available in the article.

## Abbreviations

ABA-WDS	ABA-water deficit stress domain
ADR1	activated disease resistance 1
AP2	apetala 2
Bet v 1	pathogenesis-related protein Bet v 1 family
DRM1	dormancy-associated protein 1
EXL2	exordium like 2
JMJ16	jumonji domain-containing protein 16
LRR-RLK	leucine-rich repeat receptor-like kinase
NAC	NAC transcription factor
NB-ARC	nucleotide-binding domain shared with APAF-1, various R-proteins and CED-4
Oxidored FMN	NADH:flavin oxidoreductase/NADH oxidase family
Phi 1	phosphate-induced protein 1 conserved region
Pkinase	protein kinase domain
ROS	reactive oxygen species
RPK1	receptor protein kinase 1
SAG201	senescence-associated gene 201
WRKY	WRKY transcription factor
